# Misdiagnosis of Aortic Dissection Due to Streak Artifact in the Descending Aorta

**DOI:** 10.7759/cureus.73942

**Published:** 2024-11-18

**Authors:** Takushi Santanda, Yuichi Nakamura, Joji Ito

**Affiliations:** 1 Department of Critical Care Medicine, Itabashi Chuo Medical Center, Itabashi, JPN; 2 Department of Emergency and Critical Care Medicine, Tokyo Bay Urayasu Ichikawa Medical Center, Urayasu, JPN; 3 Department of Cardiovascular Surgery, Itabashi Chuo Medical Center, Itabashi, JPN; 4 Department of Cardiovascular Surgery, Tokyo Bay Urayasu Ichikawa Medical Center, Urayasu, JPN

**Keywords:** aortic dissection, cardiac pulsation artifacts, contrast-enhanced ct, diagnostic imaging, ecg-gated ct, motion artifacts, streak artifact, traumatic aortic injury

## Abstract

Contrast-enhanced CT is a primary tool in emergency departments for diagnosing acute aortic dissection, demonstrating high sensitivity and specificity. However, artifacts such as streak artifacts can mimic aortic dissection, leading to misdiagnosis. Here, we report a case involving a 21-year-old male who sustained traumatic injuries after a motor vehicle accident. Initial contrast-enhanced CT indicated a possible localized dissection in the descending aorta. Conservative treatment was initiated under the presumption of aortic dissection. Upon re-evaluation with ECG-gated CT, the previously identified "dissection" artifact had disappeared, revealing no actual aortic injury. This case illustrates how heartbeat-induced streak artifacts, while commonly seen in the aortic root, can also manifest in the descending aorta. Our findings underscore the importance of considering artifacts in atypical cases of aortic dissection, particularly when findings are localized to areas of the aorta in close proximity to the heart. For trauma patients, while dynamic contrast-enhanced CT remains standard, ECG-gated CT should be selectively applied where motion artifacts are suspected. This case highlights the role of advanced imaging options in distinguishing between true aortic pathology and artifacts, aiding in appropriate clinical decision-making.

## Introduction

Contrast-enhanced CT is frequently utilized in emergency departments for diagnosing acute aortic dissection due to its ease of application and ability to detect complications. The sensitivity and specificity of contrast-enhanced CT are reported to be between 98% and 100% [1]. Mimics of aortic dissection include ductal remnants, motion artifacts, infundibula, streak artifacts, and various technical factors [2]. This case report presents an instance of a streak artifact that was initially misdiagnosed as an aortic dissection, localized to the descending aorta.

## Case presentation

**Figure 1:**
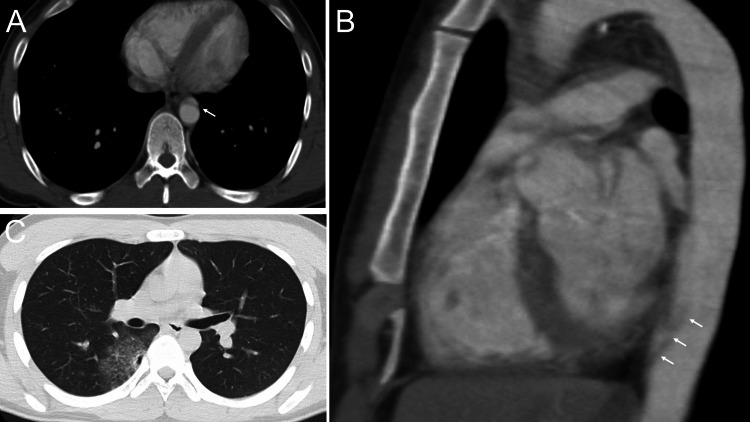
Streak Artifact in the Descending Aorta Axial (A) and sagittal (B) images of a 21-year-old man who was taken to hospital by ambulance after a traffic accident. An artifact was seen in the descending aorta where it meets the heart (arrow). It was 27 mm long. Pulmonary contusion was observed on the dorsal surface of the right middle lobe (C).

A 21-year-old male with no prior medical history was injured when he collided with a car while riding a motorbike. The patient turned right at an intersection and was struck on the left side by a car approaching at 50 km/h. He was thrown approximately 5 meters but did not lose consciousness. Upon the arrival of emergency services, he reported pain in his left shoulder and hip. In the emergency department, his vital signs were stable: he was alert, with a respiratory rate of 20/min, blood pressure of 120/97 mmHg, pulse rate of 81/min, and oxygen saturation (SpO2) at 98% on room air. Physical examination revealed only minor contusions and abrasions on the face. Contrast-enhanced CT of the chest and abdomen, performed with the patient’s arms by his side, showed an apparent localized dissection in the descending aorta posterior to the heart, as well as a traumatic pulmonary contusion (Figure 1).

**Figure 2:**
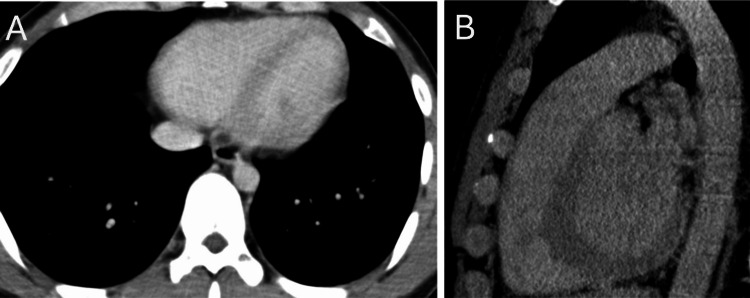
ECG-gated CT The artifacts that were recognized the day before had disappeared in the ECG-gated CT. The motion artifact has disappeared in axial (A) and sagittal (B) images.

No other abnormalities were noted. The patient was admitted with a diagnosis of traumatic aortic dissection and initiated on conservative treatment with antihypertensive and analgesic medications. As the patient’s chest pain and vital signs remained stable, cardiac CT angiography was performed the following day (Figure 2).

This subsequent CT showed no signs of aortic dissection, and the pulmonary contusion remained unchanged. The patient was discharged the next day in stable condition.

## Discussion

This case highlights the use of ECG-gated CT in clarifying a streak artifact in the descending aorta. Heartbeat-induced artifacts, typically seen as streak artifacts, usually appear in the aortic root but can occasionally extend to the descending aorta. Previous reports indicate that such streak artifacts arise from highly attenuated structures and cardiac pulsation, are often confined to one or two slices, extend beyond the aortic wall, and display a flap-like angle with variable appearance [3-5]. In this case, the artifact was present across five slices, differing from past reports, possibly due to the patient's young age, stable hemodynamics, and the improvements in imaging modalities since the 1980s and 1990s.

Dynamic contrast-enhanced CT is commonly used in emergency trauma settings [6]. However, ECG-gated CT, which is more effective in identifying motion artifacts, is recommended only in certain situations, as trauma patients frequently present with tachycardia and may struggle to hold their breath. Additionally, ECG-gated CT can compromise image quality of the lung parenchyma, ribs, and spine, suggesting that it should be selectively utilized based on the clinical scenario [7].

In the initial dynamic contrast-enhanced CT, findings appeared consistent with an aortic dissection, so further consideration of streak artifacts was overlooked. Reflecting on this case, it may have been possible to rule out the streak artifact based on the absence of intra-arterial flaps, pseudo-aneurysms, or extravasation of contrast medium, as well as the lack of adjacent hematoma near the aorta or mediastinum. Transesophageal echocardiography or MRI could have been considered for additional diagnostic clarity [8], though these were not pursued due to the availability of CT and the need for pulmonary contusion monitoring. Thus, the choice of additional diagnostic methods should be based on institutional resources and clinical objectives.

## Conclusions

Traumatic aortic dissection requires prompt evaluation. Clinicians should be particularly vigilant for potential imaging artifacts, especially when abnormalities are localized in the descending aorta near the heart. These artifacts may appear over multiple imaging slices and could lead to diagnostic confusion.
